# Obstetrics and Gynecology

**Published:** 1892-11

**Authors:** E. S. M’Kee

**Affiliations:** Cincinnati


*(To be continued.)*

---

### OBSTETRICS AND GYNECOLOGY.

BY E. S. M'KEE, M. D., CINCINNATI.

At the meeting of the Obstetrical Society May 19th, Dr. Rufus B. Hall reported a case of pyosalpinx with abscess of the ovaries, and showed specimens. The patient, aged 31 years, widow, with one child 6 years old, was referred to him by Dr. Max Koehler, of this city. The patient had complained of some pelvic pain for more than a year, yet it had not been so severe as to totally disable her from her work of dressmaking. When she applied to the physician a short time before, he recognized the tumor in the pelvis, and advised an operation, after a consultation, the speaker agreeing with him. She did not consent to the operation for about two months, continuing to go several blocks twice daily from her boarding house to her work. She grew gradually worse and the pain became more severe, so she finally agreed to the operation, which was made April 21st, and the specimens here presented removed. The left tube is as large as the index finger, and contains pus; while the ovary was enlarged to the size of a pint cup—a mere shell filled with pus. They were universally adherent, as will be observed. Just as the ligature was being placed, the patient vomited, the sac ruptured, and the greater portion of the pus was turned out, and some of it spilled in the pelvis, which was thoroughly washed out. Enough pus remains so that the specimen is as large as a tea cup. The right tube was as large as the left, and also contains pus, while the ovary is the size of an orange with pus in it, as will be observed when it is laid open before you. An interesting feature of the case is that the patient could walk and work and apparently suffer so little pain with this large quantity of pus in her pelvis. This patient weighed 165 lbs., and had the appearance of perfect health. Her case is reported to emphasize a point on which the profession is not agreed, but without which I am convinced this case would have been lost, and after which she made a rapid and complete recovery. I refer to re-opening the abdomen. After the operation everything went well for a few days. The drainage tube was removed 48 hours after the operation; the pulse ranged from 70 to 80, with a temperature from 98.8 to 99.4 until the morning of the 5th day, when the temperature was 101; yet the patient did not complain. The temperature was 101.5 in the evening, with some discomfort in left side of pelvis. The symptoms grew worse from this until the morning of the 8th day, when in spite of large and repeated doses of phenacetine her temperature was 103.6. The abdomen was re-opened and fully a pint of pus evacuated. The cavity was washed out and drained. In three hours the temperature was 100, and but once after that time did it reach that point. She made a rapid recovery. I have reason to consider it a surgeon's duty to re-open the abdomen after pus cases when similar symptoms to the case reported are presented. It is a procedure I can recommend most heartily from personal experience, and in my judgment it is not so dangerous as generally believed.

Dr. Gustav Zinke reported a case of dystocia from dislocation of the cervix during labor. The doctor reported the case not to teach the society anything, but to ask if any members of the society had met with a similar case. The patient had suffered intensely from dysmenorrhœa, but was healthy otherwise. The patient suffered from insomnia for a week before the delivery, and was given chloral, bromide of potassium and codeine, without much benefit. She suffered from pain in the back without intermission. Two days before labor set in in earnest the pains became intermittent in character. External examination led to the belief that the position was the third position of the vertex. As the pains increased, the patient became more and more restless and labor still did not come on.

Later, the pains became more regular, the patient bearing down as much as she could. On digital examination the os was found so high up that it was impossible to get the finger into it. It was pointing directly against the sacrum, and the lower segment of the uterus, very much thinned, presenting. The doctor tried to introduce two fingers, get one into os and drag it down. Although very careful to avoid rupturing the membranes, the liquor annii escaped about this time, but he could not believe it was his fault. This complicated matters and was to be regretted. Chloroform anesthesia was induced and the smallest sized Barnes dilator introduced. This remained *in situ* till six o'clock. There was no obstuction to the child's head except the undilated os. Dr. Eichberg was called in consultation. He gave chloroform, and delivery was effected with the aid of the forceps. The patient has a severe pain which has been present every since confinement. There is no odor or other bad symptoms. The highest temperature reached was 103. He believed the cause of the dislocation of the os was the contraction of the sacro-uterine ligaments. He had two cases before, which were somewhat similar to this and very slow in delivery. The patient was placed in the knee-chest posture and kept there as long as she could stand it; but this failed to correct the dislocation.

Dr. C. D. Palmer thought the condition in Dr. Zinke's case was due to a congenital elongated cervix, the so-called conoid cervix.

Dr. A. J. Miles has been successful in changing the position of the cervix, by changing the position of the patient to standing knee-chest and so forth.

Dr. A. W. Johnstone had one case where the anterior wall of the uterus presented. He thought it possible there was some trouble at the sacro-iliac junction, which, when present, is very painful.

Dr. C. A. L. Reed thought he had had similar cases to that under discussion. He could not bring himself to believe that the trouble was due to contraction of the sacro-uterine ligaments. The doctor described the action of the ligaments to be opposite in effect to that ascribed to them.

Dr. Geo. E. Jones had a case which tallied very closely to that of Dr. Zinke. He tried four days to get the os to dilate, and finally had to use forceps. He thought in the case reported that if the doctor had waited some time he would have saved himself trouble.

Dr. Zinke in closing said he did not think it a case of conical cervix, as it had all the appearances of a normal cervix, except that it had not disappeared sufficiently. To bring about a retroflexion or an anteflexion, the contraction of the ligaments must have existed for sometime. The Barnes dilators were not inserted till the membranes had ruptured.

Habitual abortion, use of asafœtida. (*Turazzo, Centrallblatt fur Gynekologie*). Good results are reported by this author from asafœtida in habitual abortion, when not dependent upon syphilis, tuberculosis or diseases of the womb or its annexes. He prescribes: R. Gum resin, asafœtida, f. pill No. lx. S. One pill every two days, gradually reducing frequency of dose.

Hypnotics for the relief of labor pains. (*Annales de Gynekologie et Obstetrique.*) Oui hypnotized an hysterical primipara after the os was dilated and succeeded in entirely suppressing all sensation of pain from subsequent uterine contractions. He introduced the hypnotic state by pressure upon the eye-balls through the closed lids. On releasing the pressure, the woman became conscious at once and felt the pains. The hypnotic effect could not be induced before dilatation of the os; after this was accomplished, it continued throughout labor without affecting the contractions.

Vomiting of pregnancy. (*Der Frauenarzt*, March, 1892). Routh states that in seven years practice, he has always been able to arrest the vomiting of pregnancy by brushing the cervix and lower cervical canal with a mixture of equal parts of iodine, iodide of potassium, spirits of wine, and water. Vomiting usually ceases immediately after application. If vomiting should recur, the cervix should again be brushed.

Parasitic fœtus. (*Archives de Tocologie.*) Leon reports a curious case. The patient, a girl 3 years old, was the child of native Mexican parents. She exhibited some well marked portions of a fœtal face on the left gluteal region. They were the upper and lower eyelids of the left eye with eyelashes and eyebrows, an upper lip which perfectly covered a part of a rudimentary upper jaw, furnished with three or four well developed incisor teeth, and a small buccal cavity with a rudimentary tongue and some fluid secretion. Near to the groove between the buttocks, was a row of silky hairs. In the inferior part of the cyst, the presence of fluid was detected, and on the surface of the parts were seen some mamillary projections.

Puerperal tetanus. (*Gazette des Hepitaux.*) Vinay holds there are three things to be achieved in the treatment of puerperal tetanus: 1. Local interference with the uterine lesion to cause elimination and destruction of the poison. 2. To change the composition of the blood altered by the poison. 3. To lessen the nervous irritability. Of all the different remedies, the inhalation of cloroform up to narcosis is the most valuable. Next to this comes chloral, which may be given in hourly 15 grain doses, either by mouth or per enemata, to be continued until sleep or muscular relaxation sets in. The subcutaneous injections of morphine may be a resort. A combination of chloral with chloroform seems to be the best treatment. Repeated warm baths with subsequent packing, act as sedatives, but are not to a danger of fresh irritation. General treatment should be very judicious. The patient should be removed from physical or mental excitement, temperature of room should be regulated, bowels regular, and nourishment strengthening. Patient should remain in bed a long time after convalescence has begun, to avoid fresh attacks.

---

AN OVERLOOKED FACTOR IN THE PRODUCTION OF CONJUNCTIVITIS.—Under this title, Dr. Julius Pohlman (*Buffalo Medical and Surgical Journal*, July, 1892) advances the idea that the effect of heat in producing conjunctivitis, is due to dryness of the atmosphere and not to its temperature. He made several experiments, and found that the disagreeable eye-symptoms, caused by hot, dry atmosphere, were at once relieved by the instillation of a drop of water. He thinks this accounts for a temperature of 70 degs. F. in a room causing conjunctivitis, when a summer heat of 95 or 100 degrees has no such effect.—*Ex.*

